# Human Health Risks Attributable to Tyre-Derived Crumb Rubber—A Literature Review on Artificial Turfs and Playgrounds

**DOI:** 10.3390/jox16050177

**Published:** 2026-09-18

**Authors:** Federica Ghelli, Giulia Squillacioti, Samar El Sherbiny, Valeria Bellisario, Roberto Bono

**Affiliations:** Department of Public Health and Pediatrics, University of Turin, via Santena 5 bis, 10126 Turin, Italy; federica.ghelli@unito.it (F.G.); samar.elsherbiny@unito.it (S.E.S.); valeria.bellisario@unito.it (V.B.); roberto.bono@unito.it (R.B.)

**Keywords:** public health, risk assessment, end-of-life tyres, artificial turfs, synthetic turf

## Abstract

Tyre granule-based artificial turf surfaces are currently a matter of debate. Most of the Agencies involved in environmental and health protection have stated a negligible/virtually negligible risk to health and have issued a general call for caution concerning the possible presence of emerging risks. The concern for the potential detrimental effect, especially in children, has attracted significant resonance since the beginning of the use of these surfaces. This review aims to describe the current knowledge in both scientific and grey literature concerning the possible adverse risks to health due to human exposure to artificial turf or playgrounds with tyre-derived crumb rubber. We included 20 original research articles and 13 reports published between 2006 and 2025. The current knowledge about the topic was revealed to be still highly fragmentary, with stark differences in methodological approaches and theoretical assumptions used in the risk assessment. However, most of the research and reports are consistent in their results, assessing, in the majority of cases, a low/negligible risk for both professional and recreational players, even though some chemicals were able to leach or volatilise and, in some cases, the estimated risk was higher than the reference threshold conventionally applied for the risk assessment method employed.

## 1. Introduction

The potential detrimental effects for living organisms associated with the surfaces incorporating crumb rubber from end-of-life tyres are still debated. Whilst, on the one hand, the possible adverse effects on the environment and human health of chemicals in the rubber infill is still a matter of debate due to the lack of data on their bioavailability [1], on the other hand, most granules and fibres can be classified as microplastics [2], i.e., solid particles smaller than 5 mm, with a high-polymer content, insoluble in water and non-degradable [3,4]. However, in a recent critical review, Sharmin et al. [5] pointed out significant gaps in current knowledge concerning the degradation rates and release of microplastics and chemicals from artificial turf.

Although most of the agencies involved in environmental and health protection assessed that most of the health risks were negligible or virtually negligible and issued a general call for caution, concern for the potential detrimental effects, especially in children, has attracted significant media attention over the past few years since the widespread use of these surfaces. Indeed, crumb rubber derived from end-of-life tyres has been employed as infill material in the USA from 1966 and in Europe from the 1980s in venues for a wide variety of sports, such as football, American football, rugby, Gaelic sports, baseball, and lacrosse [6,7] and as safety tiles for children’s play-ground areas [8]. Considering the wide variety of sports, events, and occasional activities that take place at sporting venues with artificial turf, a very high number of individuals—especially children—are regularly or occasionally exposed to these surfaces [7]. In the EU alone, the number of registered and unregistered players for football, Gaelic sports, lacrosse, and rugby was estimated to exceed 38 million individuals [7].

From a regulatory perspective, the situation worldwide is quite patchy. In Europe, the granular infill material used in sports surfaces has been considered a source of intentionally added microplastics to the environment and, after a transitional period, its use will be limited by means of a ban that will enter into force from October 2031. This measure, restricting the placing on the market of synthetic polymer microparticles, will add to the bans, restrictions, and moratoriums already in force in some US jurisdictions and communities [9]. The concern is due to the potential risks for the environment and human health of these ubiquitous particles, either synthetic or chemically modified natural, that are insoluble in water, slow to degrade and easily ingested by living organisms [10].

Tyres are complex products engineered for optimal performance, whose composition varies by type, application, and manufacturer. This results in a specific mixture of Styrene Butadiene Rubber (SBR), natural rubber, and butadiene rubber, blended with a unique combination of additives tailored to each formulation [2].

Assessing the potential risks of exposure to tyre-derived crumb rubber is thus anything but simple, since it is difficult to quantify the possible health effects, either specific or combined, as well as the particular mixture or single components responsible for them, their quantities and sources [6].

The research on the potential toxic effects associated with exposure to infill material is still ongoing, investigating several pathways, such as the potential in vitro endocrine effects [11], exposure to environmentally persistent free radicals carried onto the crumb rubber particles [12], as well as the quantification of emerging pollutants of environmental and health concern [13].

In this framework, identifying exposure levels and scenarios that can be assumed safe or tolerable is of the utmost priority. Indeed, the maximum concentration of a chemical that poses no risk of adverse effects may vary according to the circumstances, making it necessary to define stringent thresholds to protect human and environmental health [14]. Moreover, sporting venues are not isolated from surrounding contexts; some pollutants found on artificial turf surfaces may originate from sources other than crumb rubber, requiring a complex analysis of the infrastructure within the surrounding environment [15].

Hence, there is a need for a proper risk assessment, identifying hazards, quantifying relative exposures and evaluating the extent of the risks involved [16].

In our previous critical analysis, we investigated the bioaccessibility of chemicals in crumb rubber to assess their potential release in simulated biological fluids, highlighting a significant heterogeneity in both chemical concentrations and methodological approaches [1]. However, the bioaccessibility data are not per se sufficient to estimate the potential health risks for users. To bridge this gap, the present review aims to provide a comprehensive overview of the available evidence, in both scientific and grey literature, regarding the possible adverse risk to human health due to exposure to tyre-derived crumb rubber in artificial turf and playgrounds.

## 2. Materials and Methods

### 2.1. Search Strategy

The details of the search strategy used in the present review were previously described in Ghelli et al. [1]. Briefly, five databases were investigated, i.e., PubMed, Embase, CAB Direct, Scopus and Web of Science. The search was first launched on 16 May 2023 and then updated up to 1 September 2025. The search strings, previously published [1] and available in Appendix A, included keywords concerning artificial turf surfaces with tyre-derived crumb rubber (e.g., “artificial turf”, “synthetic turf”, “crumb rubber”, “infill material”, etc.) and the potential adverse effects on health (e.g., “health”, “toxicity”, “risk assessment”, “carcinogens”, “mutagens”, “mortality”). Moreover, the search was extended to the grey literature by means of a specific query on Google and Google Scholar (Appendix A) and implemented through the snowball method from the reference list in the included articles. The present review was conceived as a narrative review supported by a structured literature search to enhance its comprehensiveness and transparency of the evidence-identification process. No protocol was registered for this review.

### 2.2. Eligibility Criteria and Data Extraction

The eligibility criteria and the data extraction strategy applied were previously described in Ghelli et al. [1]. Specifically, this review includes the following: (i) original research published in the scientific literature focusing on the assessment of potential risks for human health related to exposure to crumb rubber in sporting venues and playgrounds; (ii) grey literature reports from agencies involved in environmental and human health protection evaluating the potential risks for human health based on original data or elaboration of previously recorded data related to exposure to crumb rubber in sporting venues and playgrounds. Full-text articles not reporting data or written in languages other than English were excluded, as were reviews, expert opinions, editorials, protocols, and conference papers. All records retrieved were imported into EndNote [version 21; Clarivate, Philadelphia, PA, USA] for reference management and duplicate removal. A further manual check was performed to identify and remove any remaining duplicates. Once duplicates were removed, two independent and blinded reviewers (F.G. and S.E.S.) screened the remaining records. This process was first based on title and abstract and followed by a full-text evaluation. Any disagreement was resolved by consulting a third reviewer (G.S.). Data extracted for inclusion in the present review (F.G. and S.E.S.) were as follows: country, target population, surface type, surface age and conditions, indoor/outdoor settings, exposure scenario, exposure type, exposure—pollutant classes, risk assessment method, route of exposure, main findings related to both cancer and non-cancer risk, worst-case scenario, original interpretation, study-specific benchmark, funding.

### 2.3. Visualisation and Data

A graphical abstract has been created with BioRender (https://app.biorender.com/). Data presented only in graphs in the original manuscripts and not available as numerical values in the text, tables or Supplementary Materials were extracted using the online WebPlotDigitiser software (version, 4 https://apps.automeris.io/wpd4/, accessed on 31 July 2026). The values obtained by means of this tool were visually checked against the original graphical representation to ensure consistency and properly marked in the Supplementary Materials Tables S1 and S2.

## 3. Results

### Qualitative Synthesis

**Figure 1:**
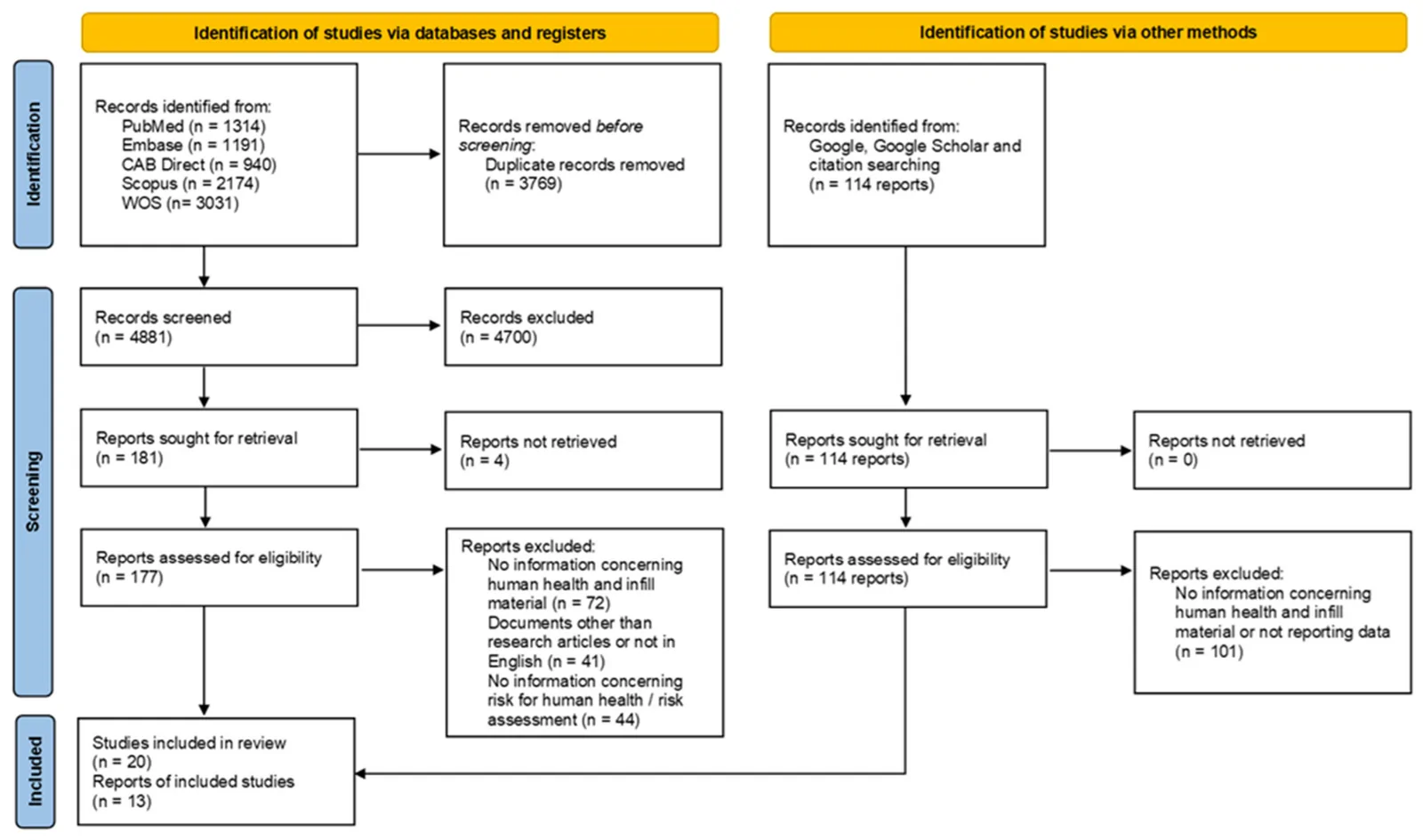
Summary of the article search strategy (PRISMA diagram [48]).

The search across five biomedical and life sciences electronic databases (i.e., PubMed, Embase, CAB, Scopus, and WOS) provided 8650 documents. After duplicate removal, the remaining 4881 items underwent a multi-step screening process according to the inclusion/exclusion criteria identified and previously described. In the end, 20 original research articles were identified [17,18,19,20,21,22,23,24,25,26,27,28,29,30,31,32,33,34,35,36]. Concerning grey literature, Google/Google Scholar provided 59 items, while screening of the included articles’ bibliographies allowed the identification of an additional 55 items. At the end of the screening process, we included 13 items [2,7,37,38,39,40,41,42,43,44,45,46,47]. To enhance the transparency of the structured search strategy, the screening process is summarised in the PRISMA flow diagram in Figure 1.

The papers and reports included at the end of the screening process were referred to several countries all over the world: China (Academic Papers (AP) *n* = 4), Denmark (Reports (Rep) *n* = 1), Egypt (AP *n* = 1), Europe (Rep *n* = 3), France (Rep *n* = 1), Germany (AP *n* = 1), Greece (AP *n* = 1), Italy (AP *n* = 3), Korea (AP *n* = 3), Netherland (AP *n* = 1, Rep *n* = 1), Norway (Rep *n* = 1), Portugal (AP *n* = 1), Turkey (AP *n* = 1), and USA (AP *n* = 4, Rep *n* = 6). The summary of the studies and the main findings are reported in Supplementary Materials (Tables S1 and S2).

**Figure 2:**
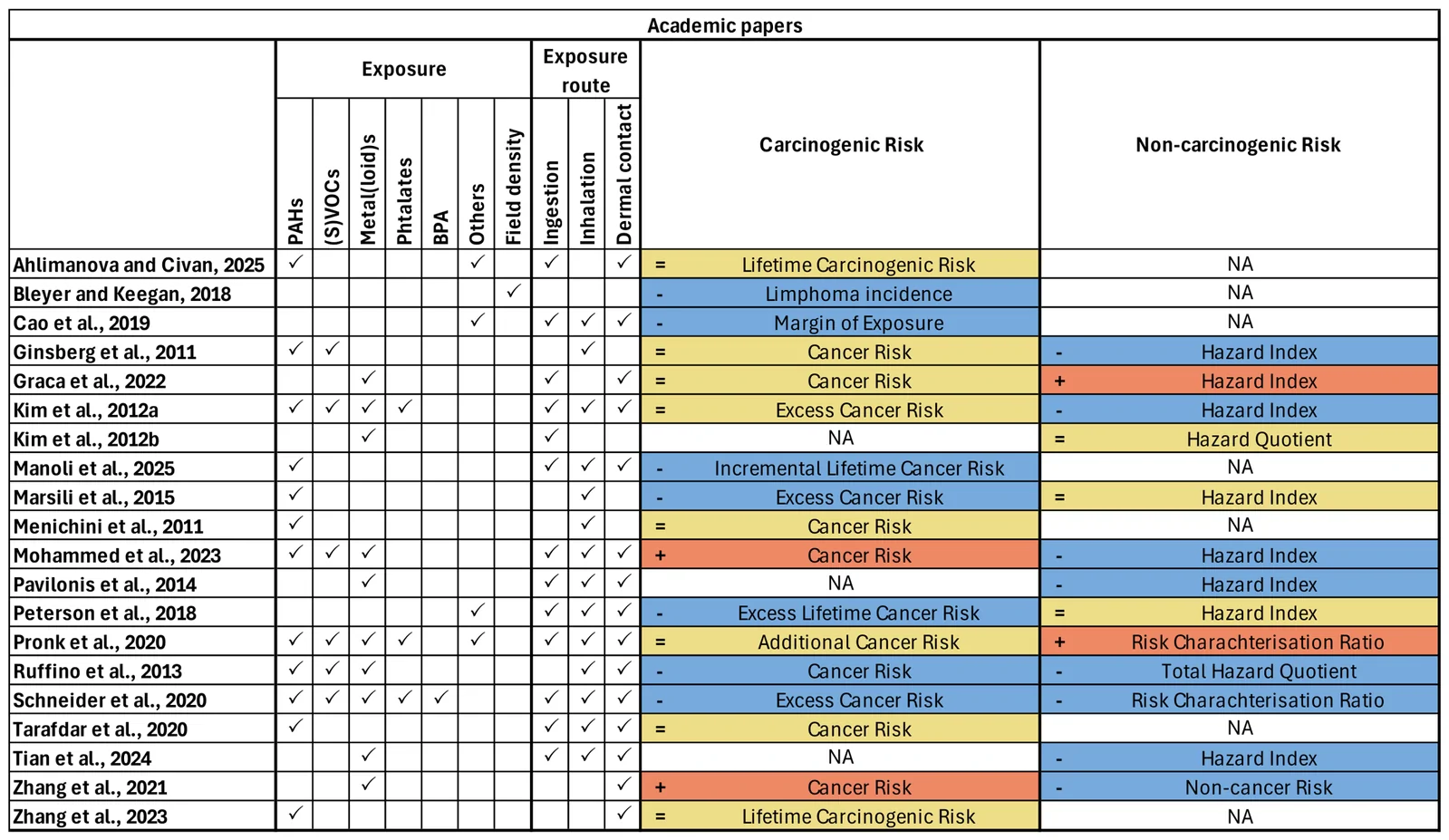
Summary of the class of chemicals, route of exposure and risk assessment method reported in academic papers. Legend: red (+): At least one of the results of the risk assessment resulted in a risk > $1\times 10^{-4}$ (cancer risk), 1 (non-carcinogenic risk), and/or margin of exposure < 1000 for all the chemicals under investigation for all the route of exposure; yellow (=): At least one of the results of the risk assessment resulted in a risk between $1\times 10^{-6}$ (cancer risk) and $1\times 10^{-4}$, close to 1 (non-carcinogenic risk), and/or margin of exposure almost 1000 for all the chemicals under investigation for all the route of exposure; blue (-): the risk assessment results were Cancer risk < $1\times 10^{-6}$ and/or HI <1 and/or margin of exposure > 1000 for all the chemicals under investigation for all the route of exposure. NA: Not Available. References of the articles cited in Figure 2 [17,18,19,20,21,22,23,24,25,26,27,28,29,30,31,32,33,34,35,36].

**Figure 3:**
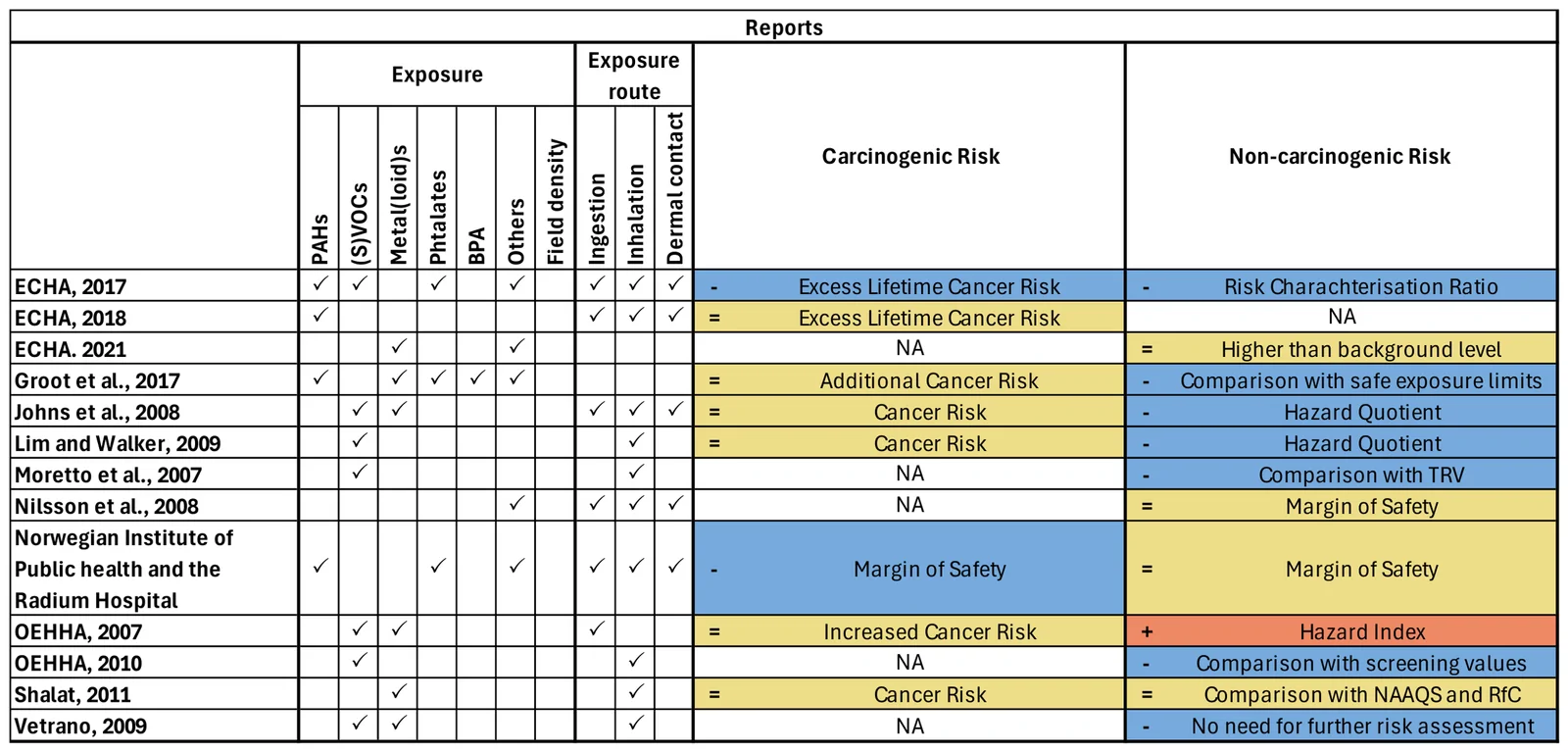
Summary of the class of chemicals, route of exposure and risk assessment method reported in reports (grey literature). Legend: red (+): At least one of the results of the risk assessment resulted in a risk > $1\times 10^{-4}$ (cancer risk), 1 (non-carcinogenic risk) or highlighted as a potential risk when compared with health-based reference values; yellow (=): At least one of the results of the risk assessment resulted in a risk between $1\times 10^{-6}$ (cancer risk) and $1\times 10^{-4}$, close to 1 (non-carcinogenic risk) or almost close to the health-based reference values; blue (-): the risk assessment results were < $1\times 10^{-6}$ (cancer risk), <1 (non-carcinogenic risk), lower than the health-based reference values for all the chemicals under investigation for all the route of exposure, or when no need for a further evaluation was assessed. NA: Not Available; NAAQS: National Ambient Air Quality Standard; RfC: Reference Concentration. References of the reports cited in Figure 3 [2,7,37,38,39,40,41,42,43,44,45,46,47].

As can be observed in Figure 2, the most investigated chemicals were PAHs (AP *n* = 12, Rep *n* = 4) and Met-al(loid)s (AP *n* = 10, Rep *n* = 6), followed by (S)VOCs (AP *n* = 6, Rep *n* = 7), Phthalates (AP *n* = 3, Rep *n* = 3), BPA (AP *n* = 1, Rep *n* = 1), and other organic chemicals (AP *n* = 4, Rep *n* = 5). Dermal contact represents the most investigated route of exposure in scientific literature (75%, *n* = 15 out of 20), while inhalation is the most studied in grey literature (77%, *n* = 10 out of 13). Concerning the scientific literature, 85% (17 out of 20) of the included articles analysed the carcinogenic risk, of which 12% (2 out of 17) highlighting a risk higher than $1\times 10^{-4}$ for at least one of the chemicals under investigation in at least one of the studied scenarios, and 47% (8 out of 17) a risk between $1\times 10^{-6}$ and $1\times 10^{-4}$; 65% (13 out of 20) of the papers investigated the non-carcinogenic risk, 15% (2 out of 13) highlighting a risk > 1 at least one of the chemicals under investigation in at least one of the studied scenarios and 23% (3 out of 20) a risk around 1. Concerning reports, 62% (8 out of 13) analysed the cancer risk, of which 75% (6 out of 8) a risk between $1\times 10^{-6}$ and $1\times 10^{-4}$ for at least one specific scenario, while 92% analysed the non-carcinogenic risk (12 out of 13), of which 8% (1 out of 12) highlighting a risk for at least one of the chemicals under investigation in at least one of the studied scenarios and 33.3% (4 out of 12) revealing a risk close to the reference benchmark, as summarised in Figure 2 and Figure 3. The classifications and percentages presented herein are strictly descriptive and must not be considered as a meta-analytic risk estimate.

The risk assessment methods employed were highly heterogeneous. In both scientific and grey literature, the authors used a wide variety of risk assessment methods, measurements, and theoretical assumptions (e.g., exposure scenarios, body weight, exposure duration and frequency, inhalation rate, ingestion rate, quantities of rubber granulate ingested per event, dermal absorption, skin exposed to the rubber granulate, risk interpretation, etc.). As an example, Johns et al. [39] used VOCs indoor measurements also for the risk assessment for outdoor fields, as a worst-case scenario, and Shalat et al. [46] presented the cancer risk for As ($1\times 10^{-5}$) as “inconsequential” even if higher than $1\times 10^{-6}$. Moreover, even when comparing the same class of chemicals, the list of chemicals under investigation was not always the same.

Some authors provided interesting results to better contextualise the exposure to chemicals in crumb rubber and the relative effects. Groot et al. [38] confirmed a small contribution of PAHs from crumb rubber compared to diet. Pavilonis et al. [28] estimated, instead, the Blood Lead Levels (BLL). Assuming the EPA gastrointestinal and lung absorption rates (30% and 32% respectively), the probability to equal or exceeding the 5 μg/dL Centers for Disease Control and Prevention (CDC) reference value, set for children between 1 and 5 years old, was less than 0.5%, whilst assuming a 100% absorption rate, the probabilities were 22.5% for children between 6 and 7 years old and 34% for children between 2 and 7 years old. It is necessary to clarify that this BLL reference value, defined in 2012, has been updated in 2021 by the CDC and reduced to 3.5 μg/dL [49].

Concerning the relationship between players’ age and risk, Ruffino et al. [31] reported a higher cancer risk for adult players due to the longest exposure. Conversely, the Authors reported a higher non-cancer risk for children compared to adults, in line with Tian et al. [34], reporting a health risk 3–5 times higher than for adults, maybe due to a more likely intake of dust and particles.

Concerning the link with the surrounding environments, Mohammed et al. [27] reported that sunlight and heat can have a role in enhancing both the cancer and non-cancer risks due to chemicals, as well as habits of artificial turf users. Lim and Walker [40] and the Norwegian Institute of Public Health and the Radium Hospital [43] pointed out that the sources of the measured chemicals may also be other than crumb rubber and come from the surrounding environment, resulting in different concentrations in upwind and downwind samples.

## 4. Discussion

There are widely differing positions concerning the use of artificial crumb rubber in sports halls and playgrounds, even in the scientific field. Most of the agencies involved in Environmental and Health protection have defined the risks for human health as negligible and proposed a general call for caution related to emerging risks. The Synthetic Turf Study developed by the California Office of Environmental Health Hazard Assessment (OEHHA), published after the date of our literature search, and therefore not included in the formal evidence synthesis, confirmed the absence of “*significant health risks to players, coaches, referees and spectators from on-field or off-field exposure to chemicals in crumb rubber infill from synthetic turf fields based on the assessment method and available data*” [50]. On the other hand, institutions such as the Children’s Environmental Health Center of the Mount Sinai School of Medicine (NY, USA), recommend the use of natural grass fields as the safest option for children’s recreational activities [51]. Although the new forthcoming regulations will result, at least in Europe, in a progressive decline in the use of artificial turf venues, the existing facilities and playgrounds will continue to be used by a significant number of individuals, as fundamental infrastructures to promote physical activity [1]. Indeed, a sedentary lifestyle is nowadays considered a global pandemic and a public health priority [52].

In 2017, the European Chemicals Agency (ECHA) identified several limitations to the estimation of the possible risks for health, such as the paucity of data concerning the characterization of the chemical profile in tyre rubber granulate, and the possible presence of substances not yet identified that could have detrimental effects. Moreover, they pointed out that many input values were based on assumptions [2]. Thereafter, the ERASSTRI project sought to clarify some of these issues; however, the authors noted that some uncertainties were still present (e.g., the absence of empirical data concerning the real exposure) and that the approach employed for the risk assessment was likely leading to an overestimation of the real risks [32]. In this framework, the computational toxicology assessment was presented as a valuable tool to identify chemicals to be prioritised, not only in association with an increase in long-term risks but also in terms of sensitisation and acute effects [53].

The main result emerging from this review is the highly fragmented nature of the existing literature. The authors of the original articles and reports employed different methods in the risk assessment and focused on different types of exposure. Considering the high heterogeneity in chemical concentration, since each manufacturer has its own specific blend for each product type, and given that the composition and bioaccessibility of chemicals are influenced over time by particle size and environmental exposure, the results obtained are difficult to compare and to generalize. Moreover, the reliance on different theoretical assumptions across the risk assessments might not actually reflect real-world circumstances.

The scenarios examined included professional, performance-oriented, and recreational users from different age groups, with, in some cases, the estimation of the exposure, and consequent risks, according to their role (field players, goalkeepers, coaches, and bystanders). Although, on the one hand, this provides a more comprehensive picture, on the other, it highlights once more the challenges in comparing the results, as they are based on different assumptions, even for similar contexts. Most of the time data concerning the exposure scenarios were interrelated or overlapping, and it was not possible to draw the specific contribution of each of them. Specifically, the frequency and duration estimated were highly variable, in some cases with distinctions according to the age and role on the pitch, especially for children and teenagers (e.g., ranging from 2 to 10 months/year, from 1 to 5 times/week, and from 1 h to 6 h per event). Given the high sensitivity of these individuals, these differences could represent a relevant bias, as well as for the ingestion rate (from 1000 mg/die in children affected by pica to 50 mg/die), the inhalation rate (highly variable in different age groups), and for the granulates ingested (from 1 g/session to 0.01 g/session). Within this framework, a comprehensive assessment would be strongly recommended to ensure a proper estimate. This will support evidence-based decision-making regarding the viability of using these infrastructures and help in identifying any necessary safeguards.

Some authors emphasized the need to adapt risk assessment models according to the specific scenarios under investigation. Specifically, Ginsberg et al. [20] pointed out the necessity to adjust the exposure concentration for different ventilation rates due to the intensity of play and for children, since the toxicity values available are derived from animals or adult humans either at rest or performing light physical activity. According to the U.S. EPA Carcinogenic Risk Assessment Supplemental Guidance for Early Life Stages, the authors also applied enhanced potency factors to all carcinogens with documented mutagenic or clastogenic properties (i.e., chloromethane, methylene chloride, benzo[a]pyrene and related carcinogenic PAHs, and benzothiazole) to account for the children’s greater vulnerability [19].

Further open points are the exposure duration and the players’ age-related characteristics, in order to critically highlight the specific risk for high-exposure groups. Indeed, professional athletes could be subject to extended exposure periods exceeding current estimates, whereas the exposure timeframe for recreational players is likely over-calculated [20].

Regarding age, the risk values were found to be higher for children than for adults, since young subjects are more sensitive to non-carcinogenic substances [31]. In most of the studies, the estimated risks were lower than the benchmark [20,23,28,29], while Mohammed et al. and Graça et al. reported the presence of a significant risk [27] or a risk worth being better investigated [21]. Kim et al. [22] assessed a significant risk in the youngest, representing the worst-case scenario since they were assumed to be affected by PICA. Ruffino et al. [31] assessed an increased cancer risk for adults and an increased risk for non-carcinogenic outcomes in children, but in both cases lower than the risk benchmark. For the playground as well, a higher risk was estimated for children than for adults.

Concerning the settings, the cancer risk estimated for indoor settings was found to be 2–3 times higher than the outdoor, even though not significant. However, indoor concentrations were used in the risk assessment as a worst-case or highly conservative scenario even when the risk was assessed for outdoor settings.

Regarding roles or activities, instead, most of the studies did not estimate an increased risk for athletes. The maximum values were identified for field players and goalkeepers. Pronk et al. [30] and Schneider et al. [32] estimated some risks higher than the benchmark (for metals and BPA, and 2-hydroxybenzothiazole, respectively), but since the results were close to threshold values, the original interpretation was not to advise a real risk for the athletes involved.

Concerning PAHs, the use of animal carcinogenicity thresholds as a toxicological reference value for PAHs in rubber granulate introduces a further element of uncertainty [30]. Moreover, the PAHs investigated may vary among studies, potentially leading to the estimation of different toxic outcomes; as well, the possible genotoxic properties of PAHs other than the eight PAHs identified by the Registration, Evaluation, Authorisation and Restriction of Chemicals (REACH) may lead to a further risk underestimation [7]. In this regard, ECHA pointed out that, to not exceed the risk threshold, the maximum possible concentration for the sum of the eight REACH PAHs in rubber granulates should be 6.5 mg/kg. Concerning the potential additional exposure to metals, the agency stated that the risk due to Cr, Ni, Se, Be, Mg, V, and Li can be considered negligible, while recommending a more refined risk assessment for Zn and Co [37]. No concern was raised for Hg, PANA and NMP, even though their concentration in artificial turf was higher than the background soil level [37].

The exposure to some of the investigated chemicals can also occur through diet. Pronk et al. [30] highlighted that the contribution of rubber granulates to Pb exposure was limited when compared with the median exposure to this metal through food in the Netherlands. Assuming a complete absorption of the bioavailable fraction, children should ingest 0.2 g of granules during each session to be exposed to the same amount of Pb [30].

As previously mentioned, crumb rubber-based surfaces are not isolated from their surroundings; instead, they are an integral part of them. Many chemicals found in granulate rubber and in the air over turfs can also be found in the soil and in the air surrounding the pitch, as well as in the leachates from grass turfs [15,27,29,31,40,43]. Indeed, playing on natural soil cannot be classified as risk-free [29], but can result in a possible exposure to dangerous chemicals (e.g., heavy metals) and to inhalable particles [54,55]. Similarly, Čakmak et al. [56] reported that the metals found in the dust upon children’s playgrounds covered with crumb rubber-based surfaces originated from the surrounding soil and from atmospheric deposition.

The dominant route of exposure is instead related to the physicochemical properties of the different compounds. Even in this case, the approaches employed were highly heterogeneous. While some authors investigated all three exposure pathways (ingestion, dermal contact, and inhalation), others focused on the dominant route(s), based on theoretical foundations. Ingestion was, in most cases, considered the main contributor, while inhalation was assumed to be negligible, especially in outdoor fields [30]. Conversely, some authors pointed out that inhalation cannot be ignored due to the proximity to the turf surface and the high respiration rate during training/matches [57]. Ginsberg et al. [20] employed a corrective factor to account for the different ventilation rates between adults and children at different ages (a 3-fold factor for the first 3 years of life and a 1.5-fold factor for children between 4 and 10 years old) [20]. Moreover, some authors implemented the exposure estimation considering also the hand-to-mouth and object-to-mouth behaviours, proper of younger children [21,23].

The quantity of rubber granulates supposed to be ingested, especially by children, during a training/match session represents an additional controversial issue. The US Environmental Protection Agency (EPA) and the Dutch National Institute for Public Health and the Environment (RIVM) suggest an amount of 0.05 g for adults and 0.2 g for children; the latter was recently refined to 0.09 g [32,58]. On the other hand, ECHA [2] suggested 0.05 g for children and 0.01 g for adults, and OEHHA [58] estimated an ingestion of up to 1 tablespoon (10.4 g). Additionally, since the chemicals’ absorption is not known for all exposure routes, the risk assessment was based on conservative assumptions. In realistic terms, exposure for football players and recreational players is much lower than assumed [30]. Rather, ingestion tends to be deliberate, with a higher prevalence among younger individuals [20].

Regarding the supposed association between exposure to artificial turf and the lymphoma incidence, which had great media resonance in the early 2000s, the studies included in this review do not support such hypotheses. The Washington State Department of Health reported that the number of cancer cases among a group of University of Washington soccer players was not higher than expected based on population cancer rates [59]. Chemicals known or suspected to be associated with this cancer type were not detectable in the granulate samples analysed or were present at levels lower than pharmacologically active concentrations, while the cancer risk, when present, was estimated to be most likely due to the presence of PAHs [30]. The association between lymphoma incidence and artificial turf density due to differences in socioeconomic status was not confirmed, as well [18]. However, these analyses may have been limited by the ecological exposure assessment, the lack of information concerning exposure timing, latency and statistical power, and the possible influence of residual confounding factors.

The four articles identified do not provide a proper risk assessment, but only some preliminary elements and elaboration; however, they reported some interesting insights. Concerning inhalation of PAHs, Armada et al. [60] estimated the chronic exposure for a 70 kg athlete was between 0.06 and 0.45 μg/kg bw BaP_eq_ and the increased lifetime concentration for BaP was estimated to be 0.025 ng/m^3^, equal to the 2.5% of the European Target Value), whilst the daily uptake of PAHs from crumb rubber was estimated between 0.16 and 0.90% of the RfD; Birkholz et al. [61] revealed no in vitro toxicological effects, Perkins et al. [53] reported an in silico evaluation of the properties of a list of chemicals in crumb rubber, revealing that 197 out of 306 compounds were predicted to have a carcinogenic potential, of which 52 already classified as known, presumed or suspected human carcinogens by US EPA or ECHA; Huang et al. [12] reported an increase in the presence of crumb rubber levels in saliva after exercise on artificial turf fields, and >75% of the particles were <10 µm. The authors speculated that the inhalation of these particles can result in increased exposure to environmentally persistent free radicals.

Depending on their size and polymeric composition, tyre-derived infill granules may fall within current definitions of microplastics. Nevertheless, the available evidence has focused predominantly on chemical release; the potential particle-related toxicological effects still remain mainly unexplored.

The novelty of this work lies in the attempt to not only summarise the current knowledge related to the possible health risks due to exposure to tyre-based crumb rubber, but also to highlight their heterogeneity and the potential biases affecting the estimates and preventing the comparison of risk assessments. This was meant to fill the lack of an organic presentation of the results, which to date have been scattered among the scientific and grey literature; to bridge this gap, we thus included both sources. However, we have to point out that, given the narrative nature of this review, any comparison among the results falls outside the aim of this manuscript. For the same reason, a formal methodological quality assessment was not pre-planned. Our summary, specifically that provided in Figure 2 and Figure 3, was related to a classification of the numerical estimation provided by each study according to the US EPA guidelines. As can be observed in Tables S1 and S2, this may result in a more cautious classification of any risks associated with exposure to crumb rubber, highlighting how certain conditions—which the original authors had classified as posing low or negligible risks due to the use of highly conservative models or worst-case scenarios—also warrant further investigation. Our decision was based on adopting a more conservative approach, given the considerable heterogeneity of the data and the methods used to calculate these estimates. Regarding the carcinogenic risk assessment, several times it was not a matter of exceeding or not exceeding the acceptable cancer risk threshold, but rather the results fell in the area of risk management.

However, the results from both scientific and grey literature were mostly consistent, estimating, in most instances, no sufficient reasons to be concerned about the use of tyre-derived crumb rubber surfaces, even if some critical circumstances were identified, mainly related to the worst-case scenarios. The synthesis of these findings should be considered cautiously. Indeed, the methodological differences among the studies, as well as the incomplete characterisation of mixtures and emerging contaminants, the limited empirical exposure data and the very little epidemiological evidence represent undoubtedly limitations in drawing conclusions. Moreover, the lack of data concerning hazard identification, environmental concentration, estimated exposure, modelled risk, and observed health outcomes in some/most of the studies or their presentation in a non-comparable manner further increases the difficulties in deriving unequivocal results and warrants a cautious interpretation.

## 5. Conclusions

Despite the high resonance associated with the potentially dangerous effects linked to the use of artificial turf and the concern for health safety, the knowledge about the topic is still highly fragmentary. Most of the research and reports are consistent in their results, assessing, in the majority of cases, a low/negligible estimated health risk under typical exposure conditions. However, some studies reported estimates higher than the reference threshold conventionally applied for the risk assessment method employed, since some chemicals were able to leach or volatilise, or could be bioavailable. Although these results indicating a potential concern were generally related to specific exposure scenarios or worst-case scenarios and cannot be readily generalised, their possible occurrence should not be dismissed. Caution should be warranted given the complexity and variability of the exposure pathways, together with the substantial methodological limitations and uncertainties characterizing the available evidence, such as uncertain source attribution, the presence of sparse empirical exposure data, incomplete characterisation of mixtures and of chemicals originating as a result of exposure to environmental conditions, extreme variability in the original tyre mixture composition, and lack of health-outcome studies. Further research should bridge these gaps, providing an extensive and comprehensive analysis of the topic, prioritising the collection of empirical data concerning both the exposure assessment and the possible long-term outcomes, with a special focus on vulnerable groups. This holistic approach will allow the general population and stakeholders to carry out a well-informed cost-benefit assessment of using these structures based on objective data.

## Data Availability

No new data were created or analysed in this study. Data sharing is not applicable to this article.

## Supplementary Materials

The following supporting information can be downloaded at: https://www.mdpi.com/article/10.3390/jox16050177/s1. Table S1: Studies providing data concerning risk assessment related to the exposure to tyre-derived crumb rubber. Table S2: Reports providing data concerning risk assessment related to the exposure to tyre-derived crumb rubber.

## Abbreviations

The following abbreviations are used in this manuscript:

2-MBT	2-mercaptobenzothiazole
As	Arsenic
BaP	Benzo[a]pyrene
BDE	Brominated diphenyl ether
Be	Beryllium
BLL	Blood-Lead Levels
BPA	Bisphenol A
Cd	Cadmium
CDC	Centers for Disease Control and Prevention
Co	Cobalt
COPC	Chemical of Potential Concern
Cr	Chromium
CR	Cancer Risk
Cu	Copper
ECHA	European Chemicals Agency
ECR	Excess Cancer Risk
EPA	Environmental Protection Agency
Hg	Mercury
HI	Hazard Index
HQ	Hazard Quotient
ICR	Increased Cancer Risk
ILCR	Incremental Lifetime Cancer Risk
Li	Lithium
MCCPs	Medium Chain Chlorinated Paraffins
Mg	Magnesium
MOE	Margin of Exposure
MOS	Margin Of Safety
Mn	Manganese
NAAQS	National Ambient Air Quality Standard
Ni	Nickel
NMP	N-Methyl-2-pyrrolidone
OEHHA	California Office of Environmental Health Hazard Assessment
PAEs	Phthalic Acid Esters
PAHs	Polycyclic Aromatic Hydrocarbons
PANA	N-phenyl-alpha-naphthylamine
POPs	Persistent Organic Pollutants
Pb	Lead
RCR	Risk Characterisation Ratio
REACH	Registration, Evaluation, Authorisation and Restriction of Chemicals
RfC	Reference Concentration
RfD	Reference Dose
RIVM	National Institute for Public Health and the Environment
SBR	Styrene Butadiene Rubber
SCCPs	Short Chain Chlorinated Paraffins
Sb	Antimony
Se	Selenium
(S)VOCs	(Semi-)Volatile Organic Compounds
THQ	Total Hazard Quotient
TOSHI	Target-Organ-Specific Hazard Index
Tl	Thallium
V	Vanadium
Zn	Zinc

## Appendix A

**Search strings**

**PubMed**

#1 “artificial turf*”[tiab] OR “synthetic turf*”[tiab] OR “artificial grass”[tiab] OR “synthetic grass”[tiab]

#2 “crumb rubber”[tiab] OR “tire crumb”[tiab] OR “tyre crumb”[tiab:~0] OR “shredded tire*”[tiab] OR “shredded tyre*”[tiab] OR “recycled tire*”[tiab] OR “recycled tyre*”[tiab] OR “scrap tire*”[tiab] OR “scrap tyre*”[tiab] OR “tire wear”[tiab] OR “tyre wear”[tiab] OR “poured rubber”[tiab] OR “rubber granulate*”[tiab] OR “rubber granule*”[tiab] OR “tire rubber”[tiab] OR “tyre rubber”[tiab] OR “tire particle*”[tiab] OR “tyre particle*”[tiab] OR “tire debris”[tiab] OR “tyre debris”[tiab:~0]

#3 (“Parks, Recreational”[Mesh] OR playground*[tiab] OR “play area*”[tiab]) AND (“Rubber”[Mesh] OR rubber[tiab] OR infill[tiab] OR tire[tiab] OR tires[tiab] OR tyre[tiab] OR tyres[tiab])

#4 #1 OR #2 OR #3

#5 “Health”[Mesh] OR “Public Health”[Mesh] OR “Health Surveys”[Mesh] OR health*[tiab]

#6 “toxicity”[subheading] OR “Toxicity Tests”[MeSH] OR toxic*[tiab] OR neurotoxic*[tiab] OR cytotoxic*[tiab] OR reprotoxic*[tiab] OR genotoxic*[tiab] OR immunotoxic*[tiab]

#7 “Ecotoxicology”[Mesh] OR ecotoxic*[tiab] OR toxin*[tiab]

#8 “adverse effects”[subheading]

#9 “Risk Assessment”[Mesh] OR risk*[tiab]

#10 “Hazardous Substances”[MeSH] OR hazard*[tiab] OR harm*[tiab] OR concern[tiab] OR concerns[tiab]

#11 “Carcinogens, Environmental”[Mesh] OR carcinogen*[tiab]

#12 “Mutagens”[Mesh] OR mutagen*[tiab]

#13 “Environmental Exposure”[MeSH] OR “Environmental Health”[Mesh] OR “Environmental Monitoring”[Mesh] OR expos*[tiab] OR monitor*[tiab] OR surveill*[tiab]

#14 “Incidence”[Mesh] OR incidence[tiab]

#15 “Prevalence”[Mesh] OR prevalence[tiab]

#16 “epidemiology”[Subheading] OR epidemiol*[tiab]

#17 “Mortality”[Mesh] OR mortality[tiab]

#18 bio-accessibility[tiab] OR bioaccessibility[tiab]

#19 “Fluids and Secretions”[Mesh] OR bio-fluid*[tiab] OR biofluid*[tiab] OR blood[tiab] OR saliva[tiab] OR sweat[tiab] OR tears[tiab] OR urine[tiab]

#20 #5 OR #6 OR #7 OR #8 OR #9 OR #10 OR #11 OR #12 OR #13 OR #14 OR #15 OR #16 OR #17 OR #18 OR #19

#21 #4 AND #20

**Embase**

#1 ‘artificial turf’/exp OR ‘artificial turf*’:ti,ab,kw OR ‘synthetic turf*’:ti,ab,kw OR ‘artificial grass’:ti,ab,kw OR ‘synthetic grass’:ti,ab,kw

#2 ‘crumb rubber’/exp OR ‘crumb rubber’:ti,ab,kw OR ‘tire crumb’:ti,ab,kw OR ‘tyre crumb’:ti,ab,kw OR ‘shredded tire*’:ti,ab,kw OR ‘shredded tyre*’:ti,ab,kw OR ‘recycled tire*’:ti,ab,kw OR ‘recycled tyre*’:ti,ab,kw OR ‘scrap tire*’:ti,ab,kw OR ‘scrap tyre*’:ti,ab,kw OR ‘tire wear particle’/exp OR ‘tire wear’:ti,ab,kw OR ‘tyre wear’:ti,ab,kw OR ‘poured rubber’:ti,ab,kw OR ‘rubber granulate*’:ti,ab,kw OR ‘rubber granule*’:ti,ab,kw OR ‘tire rubber’:ti,ab,kw OR ‘tyre rubber’:ti,ab,kw OR ‘tire particle*’:ti,ab,kw OR ‘tyre particle*’:ti,ab,kw OR ‘tire debris’:ti,ab,kw OR ‘tyre debris’:ti,ab,kw

#3 (‘recreational park’/exp OR playground*:ti,ab,kw OR ‘play area*’:ti,ab,kw) AND (‘rubber’/exp OR ‘motor vehicle tire’/exp OR rubber:ti,ab,kw OR infill:ti,ab,kw OR tire:ti,ab,kw OR tires:ti,ab,kw OR tyre:ti,ab,kw OR tyres:ti,ab,kw)

#4 #1 OR #2 OR #3

#5 ‘health’/exp OR ‘public health’/exp OR ‘epidemiological surveillance’/exp OR health*:ti,ab,kw

#6 ‘toxicity’/exp OR ‘toxicity testing’/exp OR toxic*:ti,ab,kw OR neurotoxic*:ti,ab,kw OR cytotoxic*:ti,ab,kw OR reprotoxic*:ti,ab,kw OR genotoxic*:ti,ab,kw OR immunotoxic*:ti,ab,kw

#7 ‘ecotoxicology’/exp OR ecotoxic*:ti,ab,kw OR toxin*:ti,ab,kw

#8 ‘risk assessment’/exp OR risk*:ti,ab,kw

#9 ‘hazard’/exp OR hazard*:ti,ab,kw OR harm*:ti,ab,kw OR concern:ti,ab,kw OR concerns:ti,ab,kw

#10 ‘carcinogen’/de OR carcinogen*:ti,ab,kw

#11 ‘mutagenic agent’/de OR mutagen*:ti,ab,kw

#12 ‘exposure’/exp OR ‘environmental health’/exp OR ‘environmental monitoring’/exp OR expos*:ti,ab,kw OR monitor*:ti,ab,kw OR surveill*:ti,ab,kw

#13 ‘incidence’/exp OR incidence:ti,ab,kw

#14 ‘prevalence’/exp OR prevalence:ti,ab,kw

#15 epidemiology:lnk OR epidemiol*:ti,ab,kw

#16 ‘mortality’/exp OR mortality:ti,ab,kw

#17 bio-accessibility:ti,ab,kw OR bioaccessibility:ti,ab,kw

#18 ‘biofluid’/exp OR ‘body fluids and secretions’/exp OR bio-fluid*:ti,ab,kw OR biofluid*:ti,ab,kw OR blood:ti,ab,kw OR saliva:ti,ab,kw OR sweat:ti,ab,kw OR tears:ti,ab,kw OR urine:ti,ab,kw

#19 #5 OR #6 OR #7 OR #8 OR #9 OR #10 OR #11 OR #12 OR #13 OR #14 OR #15 OR #16 OR #17 OR #18

#20 #4 AND #19

**CAB Direct**

#1 title:(“artificial turf*” OR “synthetic turf*” OR “artificial grass” OR “synthetic grass” OR “crumb rubber” OR “tire crumb” OR “tyre crumb” OR “shredded tire*” OR “shredded tyre*” OR “recycled tire*” OR “recycled tyre*” OR “scrap tire*” OR “scrap tyre*” OR “tire wear” OR “tyre wear” OR “poured rubber” OR “rubber granulate*” OR “rubber granule*” OR “tire rubber” OR “tyre rubber” OR “tire particle*” OR “tyre particle*” OR “tire debris” OR “tyre debris”) OR ab:(“artificial turf*” OR “synthetic turf*” OR “artificial grass” OR “synthetic grass” OR “crumb rubber” OR “tire crumb” OR “tyre crumb” OR “shredded tire*” OR “shredded tyre*” OR “recycled tire*” OR “recycled tyre*” OR “scrap tire*” OR “scrap tyre*” OR “tire wear” OR “tyre wear” OR “poured rubber” OR “rubber granulate*” OR “rubber granule*” OR “tire rubber” OR “tyre rubber” OR “tire particle*” OR “tyre particle*” OR “tire debris” OR “tyre debris”)

#2 subject:(“playgrounds”) OR title:(playground* OR “play area*”) OR ab: :(playground* OR “play area*”)

#3 subject:(rubber OR tyres) OR title:(rubber OR infill OR tire OR tires OR tyre OR tyres) OR ab:(rubber OR infill OR tire OR tires OR tyre OR tyres)

#4 #2 AND #3

#5 #1 OR #4

#6 subject:(“health” OR “public health”) OR title:(health*) OR ab:(health*)

#7 subject:(“toxicity”) OR title:(toxic* OR neurotoxic* OR cytotoxic* OR reprotoxic* OR genotoxic* OR immunotoxic* OR ecotoxic* OR toxin*) OR ab:(toxic* OR neurotoxic* OR cytotoxic* OR reprotoxic* OR genotoxic* OR immunotoxic* OR ecotoxic* OR toxin*)

#8 subject:(“adverse effects”)

#9 subject:(“risk assessment”) OR title:(risk*) OR ab:(risk*)

#10 subject:(“hazards”) OR title:(hazard* OR harm* OR concern OR concerns) OR ab:(hazard* OR harm* OR concern OR concerns)

#11 subject:(“carcinogens”) OR title:(carcinogen*) OR ab:(carcinogen*)

#12 subject:(“mutagens”) OR title:(mutagen*) OR ab:(mutagen*)

#13 subject:(“exposure” OR “environmental health”) OR title:(expos* OR monitor* OR surveill*) OR ab:(expos* OR monitor* OR surveill*)

#14 subject:(“incidence”) OR title:(incidence) OR ab:(incidence)

#15 subject:(“disease prevalence”) OR title:(prevalence) OR ab:(prevalence)

#16 subject:(“epidemiology”) OR title:(epidemiol*) OR ab:(epidemiol*)

#17 subject:(“mortality”) OR title:(mortality) OR ab:(mortality)

#18 title:(“bio-accessibility” OR bioaccessibility) OR ab:(“bio-accessibility” OR bioaccessibility)

#19 subject:(“body fluids” OR “secretions”) OR title:(“bio-fluid*” OR biofluid* OR blood OR saliva OR sweat OR tears OR urine) OR ab:(“bio-fluid*” OR biofluid* OR blood OR saliva OR sweat OR tears OR urine)

#20 #6 OR #7 OR #8 OR #9 OR #10 OR #11 OR #12 OR #13 OR #14 OR #15 OR #16 OR #17 OR #18 OR #19

#21 #5 AND #20

**Scopus**

#1 TITLE-ABS-KEY(“artificial turf*” OR “synthetic turf*” OR “artificial grass” OR “synthetic grass”)

#2 TITLE-ABS-KEY(“crumb rubber” OR “tire crumb” OR “tyre crumb” OR “shredded tire*” OR “shredded tyre*” OR “recycled tire*” OR “recycled tyre*” OR “scrap tire*” OR “scrap tyre*” OR “tire wear” OR “tyre wear” OR “poured rubber” OR “rubber granulate*” OR “rubber granule*” OR “tire rubber” OR “tyre rubber” OR “tire particle*” OR “tyre particle*” OR “tire debris” OR “tyre debris”)

#3 TITLE-ABS-KEY(playground* OR “play area*”) AND TITLE-ABS-KEY(rubber OR infill OR tire OR tires OR tyre OR tyres)

#4 #1 OR #2 OR #3

#5 TITLE-ABS-KEY(health*)

#6 TITLE-ABS-KEY(toxic* OR neurotoxic* OR cytotoxic* OR reprotoxic* OR genotoxic* OR immunotoxic* OR ecotoxic* OR toxin*)

#7 TITLE-ABS-KEY(risk*)

#8 TITLE-ABS-KEY(hazard* OR harm* OR concern OR concerns)

#9 TITLE-ABS-KEY(carcinogen* OR mutagen*)

#10 TITLE-ABS-KEY(expos* OR monitor* OR surveill*)

#11 TITLE-ABS-KEY(incidence OR prevalence OR epidemiol* OR mortality)

#12 TITLE-ABS-KEY(bio-accessibility OR bioaccessibility)

#13 TITLE-ABS-KEY(bio-fluid* OR biofluid* OR blood OR saliva OR sweat OR tears OR urine)

#14 #5 OR #6 OR #7 OR #8 OR #9 OR #10 OR #11 OR #12 OR #13

#15 #4 AND #14

#16 INDEX(Medline OR Embase)

#17 PMID(1* OR 2* OR 3* OR 4* OR 5* OR 6* OR 7* OR 8* OR 9* OR 0*)

#18 #16 OR #17

#19 #15 AND NOT #18

**Web of Science**

#1 TS=(“artificial turf*” OR “synthetic turf*” OR “artificial grass” OR “synthetic grass”)

#2 TS=(“crumb rubber” OR “tire crumb” OR “tyre crumb” OR “shredded tire*” OR “shredded tyre*” OR “recycled tire*” OR “recycled tyre*” OR “scrap tire*” OR “scrap tyre*” OR “tire wear” OR “tyre wear” OR “poured rubber” OR “rubber granulate*” OR “rubber granule*” OR “tire rubber” OR “tyre rubber” OR “tire particle*” OR “tyre particle*” OR “tire debris” OR “tyre debris”)

#3 TS=(playground* OR “play area*”) AND TS=(rubber OR infill OR tire OR tires OR tyre OR tyres)

#4 #1 OR #2 OR #3

#5 TS=health*

#6 TS=(toxic* OR neurotoxic* OR cytotoxic* OR reprotoxic* OR genotoxic* OR immunotoxic* OR ecotoxic* OR toxin*)

#7 TS=risk*

#8 TS=(hazard* OR harm* OR concern OR concerns)

#9 TS=(carcinogen* OR mutagen*)

#10 TS=(expos* OR monitor* OR surveill*)

#11 TS=(incidence OR prevalence OR epidemiol* OR mortality)

#12 TS=(bio-accessibility OR bioaccessibility)

#13 TS=(bio-fluid* OR biofluid* OR blood OR saliva OR sweat OR tears OR urine)

#14 #5 OR #6 OR #7 OR #8 OR #9 OR #10 OR #11 OR #12 OR #13

#15 #4 AND #14.

**Google/Scholar:**

**“artificial turf”|”synthetic turf”|”crumb rubber”|”tire crumb”|”tyre crumb” site:.gov** (Google)

**“artificial turf”|”synthetic turf”|”crumb rubber”|”tire crumb”|”tyre crumb” site:.eu** (Google)

**“artificial turf”|”synthetic turf”|”crumb rubber”|”tire crumb”|”tyre crumb” site:.nhs.uk** (Google)

**“artificial turf”|”synthetic turf”|”crumb rubber”|”tire crumb”|”tyre crumb” report (Scholar)**

Stringa Policy Commons (https://policycommons.net/):

**title:‘artificial turf’ OR title:‘synthetic turf’ OR title:‘crumb rubber’ OR title:‘tire crumb’ AND filter: reports OR white papers**
